# 

**Published:** 1908-01-11

**Authors:** 


### THE GRADUATES' MEDICAL SCHOOLS.

### Diary for the week January 13 to 19.

**THE THROAT HOSPITAL, Golden Square, W.**

**Lectures at 5.30 p.m.**

- **January 13,** Mr. T. J. Faulder, **Anatomy and Physiology of the Ear,**
- **January 16,** Mr. C. A. Parker, **Examination of the Ear and Aural Therapeutics.**

**MEDICAL GRADUATES' COLLEGE AND POLYCLINIC, Chenies Street, W.C.**

**Lectures at 5.15 p.m.**

- **January 13,** Mr. A. Carless, **Some Internal Derangements of the Knee Joint.**
- **January 14,** Dr. T. C. Stevens, **The Diagnosis and Treatment of Acute Abdominal Conditions originating in the Genital Organs.**
- **January 15,** Dr. Charles Mercier, **Functional Disease and its Treatment.**
- **January 16,** Sir Almroth Wright, **Some Points in Connection with Therapeutic Inoculation.**

**THE POST-GRADUATE COLLEGE, West London Hospital, Hammersmith, W.**

**Daily at 2:** Medical and Surgical Clinics and *x*-Rays at **2.30:** Operations.

**Special Departments at 10** (Tuesday, Wednesday, and Saturday), and also daily at **2 p.m.**

**Lectures:**

- **January 13, 12 noon,** Dr. Low, **Pathological Demonstration.**
- **January 14 and 16,** Dr. Pritchard, **Practical Medicine.**

**At 5 p.m.**

- **January 13,** Dr. Saunders, **Clinical: with Cases.**
- **January 14,** Dr. Low, **Filariasis.**
- **January 15,** Dr. Beddard, **Practical Medicine.**
- **January 16,** Mr. Baldwin, **Practical Surgery.**
- **January 17,** Dr. Kenneth Scott.

**NATIONAL HOSPITAL FOR THE PARALYSED AND EPILEPTIC, Queen Square, W.C.**

**Lectures at 3.30 p.m.**

- **January 14,** Dr. Beevor, **Arterial supply to the Human Brain, with Demonstrations of Injected Specimens.**

THE HOSPITAL *January* 11, 1908.

*Name* ..............................................................

*Address* ..............................................................

..............................................................

This Coupon must accompany manuscript or contributions intended for The Hospital.

